# When Is It Safe to Introduce an AI System Into Healthcare? A Practical Decision Algorithm for the Ethical Implementation of Black‐Box AI in Medicine

**DOI:** 10.1111/bioe.70032

**Published:** 2025-09-18

**Authors:** Jemima Winifred Allen, Dominic Wilkinson, Julian Savulescu

**Affiliations:** ^1^ Department of Paediatrics, Faculty of Medicine, Nursing and Health Sciences Monash University Clayton Victoria Australia; ^2^ Uehiro Oxford Insitute University of Oxford Oxford UK; ^3^ Newborn Care Oxford University Hospitals NHS Foundation Trust Oxford UK; ^4^ Centre for Biomedical Ethics, Yong Loo Lin School of Medicine National University of Singapore Singapore; ^5^ Murdoch Children's Research Institute Melbourne Victoria Australia

**Keywords:** artificial intelligence, black‐box AI, clinical practice, informed consent, large language models, risk assessment

## Abstract

There is mounting global interest in the revolutionary potential of AI tools. However, its use in healthcare carries certain risks. Some argue that opaque (‘black box’) AI systems in particular undermine patients' informed consent. While interpretable models offer an alternative, this approach may be impossible with generative AI and large language models (LLMs). Thus, we propose that AI tools should be evaluated for clinical use based on their implementation risk, rather than interpretability. We introduce a practical decision algorithm for the clinical implementation of black‐box AI by evaluating its risk of implementation. Applied to the case of an LLM for surgical informed consent, we assess a system's implementation risk by evaluating: (1) technical robustness, (2) implementation feasibility and (3) analysis of harms and benefits. Accordingly, the system is categorised as minimal‐risk (standard use), moderate‐risk (innovative use) or high‐risk (experimental use). Recommendations for implementation are proportional to risk, requiring more oversight for higher‐risk categories. The algorithm also considers the system's cost‐effectiveness and patients' informed consent.

## 1 Introduction

There is mounting global interest in the revolutionary potential of AI systems in medicine, like Consent‐GPT (Box 1). Yet, despite evidence suggesting that some AI‐based tools perform equivalent (if not superior) to existing approaches to patient care [1, 2], some are concerned about the uninterpretable “black‐box” nature of these systems [3, 4]. For healthcare, in particular, the use of black‐box AI could undermine patients' informed consent [5]. As a result of these (and other) ethical concerns (Box 2), many areas of medicine have been slow to adopt AI‐based tools into practice.

Box 1 **Consent‐GPT—Case introduction.**

*A group of clinicians and hospital administrators are considering implementing a novel AI tool, called Consent‐GPT* [6], *as part of the hospital's informed consent process for surgical procedures. Consent‐GPT uses artificial intelligence (AI) trained on clinically accurate datasets to interact virtually with patients in the weeks before surgery. It is designed to augment existing consent processes by streamlining clinical workflow and allowing patients more time for informed decision‐making*.

*However, some staff are concerned about the novelty of Consent‐GPT and whether current evidence is sufficient to justify its use clinically, particularly regarding the medical accuracy of its outputs, the risk of hallucinations and the technical feasibility of implementing it into existing IT systems. They also worry about how much human oversight will be needed, at least initially, and whether this will add to their already burdensome workload*.

*How should the hospital implement Consent‐GPT into its practice? What factors should influence their decision to ensure that the process is carried out ethically and responsibly?*

Box 2 **Ethical concerns relating to the use of AI in medicine [7].**

- *Difficulty in assessing the effectiveness and reliability of adaptable AI systems;*
- *Impact of biased or missing data on AI system's performance across different subgroups, potentially perpetuating historic patterns of discrimination;*
- *AI system's need for big data may make it difficult to predict future uses of data, which risks breaching patient privacy and confidentiality;*
- *AI systems require set values for programming and thus lack consideration of individual patient values (machine paternalism), which may undermine respect for patients’ autonomy;*
- *The use of AI raises moral concerns about who will be responsible if harm occurs to patients;*
- *Users of AI may lack trust in AI‐generated recommendations;*
- *Ability of AI systems to provide explanations to improve the extent to which humans can trust these systems;*
- *The use of AI in medicine risks dehumanisation and deskilling of the profession;*
- *The potential for misuse of AI leading to large‐scale catastrophic harm*.

Bridging the so‐called AI chasm [8] between AI research and its real‐world clinical application remains a major challenge for policymakers and practitioners.

To address black‐box concerns, some recommend the use of interpretable AI systems (i.e., restricting models so that humans can easily understand their outputs) to promote transparency and accountability [9]. However, others argue that transparency is not as important as ensuring that these models produce accurate and reliable clinical outputs [7, pp. 150–158].

Regardless, given the widespread availability and general utility of AI‐based tools, some are already beginning to use them, including within clinical practice [10, 11]. A lack of clear guidance risks *ad hoc* application of AI systems in healthcare, without proper human oversight or clinical evaluation [12]. Thus, there is a need for an ethical framework to ensure that currently available AI systems can be implemented into clinical practice responsibly.

**Figure 1:**
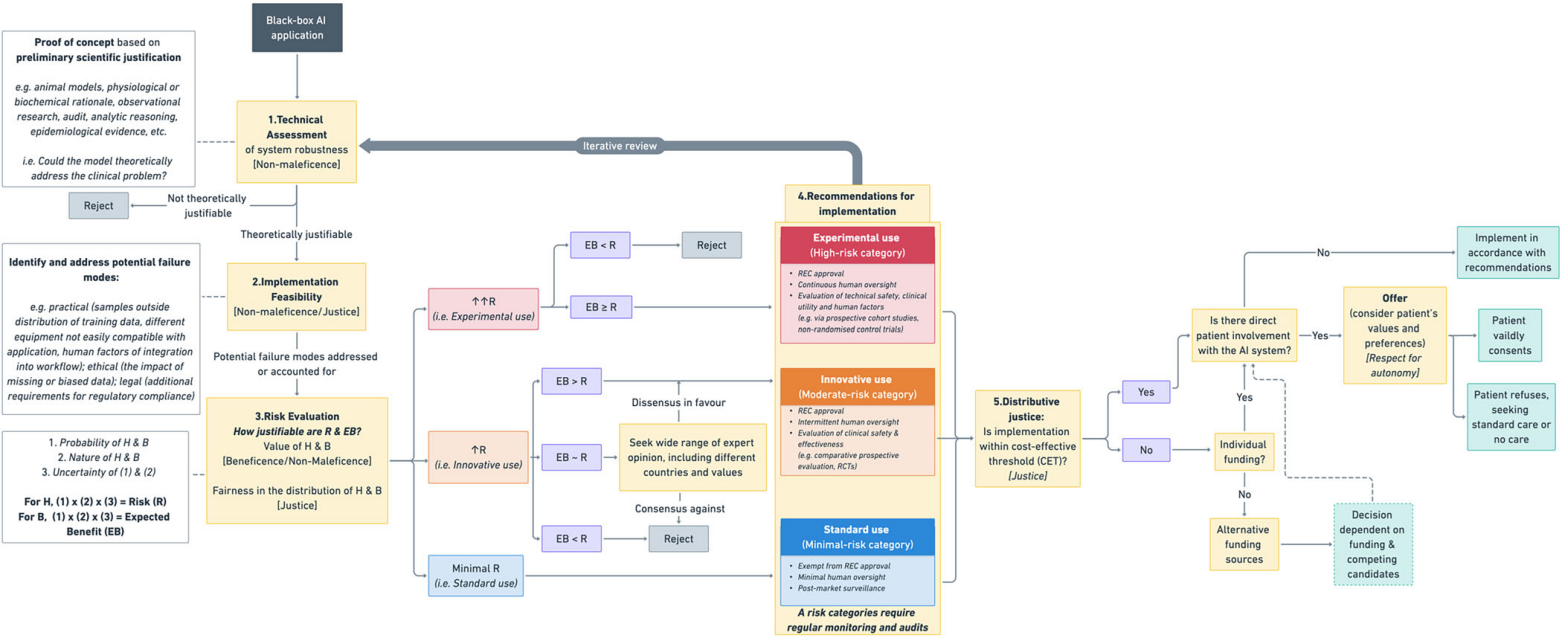
*Implementation Risk* Algorithm—A practical decision algorithm for the ethical implementation of AI systems in medicine. Black‐box AI applications undergo (1) technical assessment of system robustness [Non‐maleficence], (2) implementation feasibility assessment [Non‐maleficence/Justice] and (3) risk evaluation of harms and benefits [Beneficence/Non‐maleficence]. AI applications are then categorised into minimal‐risk (standard use), moderate‐risk (innovative use) and high‐risk (experimental use). Recommendations for implementation are proportional to risk. Finally, the application is assessed for its cost‐effectiveness and the need for patients’ informed consent. B, benefit; CET, within cost‐effectiveness threshold; H, harm; REC, Research Ethics Committee; RCT, randomised control trial. Uncertainty = 1/Confidence; Confidence ⊆ [0,1].

In this paper, we propose a practical algorithm to guide the clinical implementation of AI systems based on their associated risks (i.e., a model's *Implementation Risk*) (Figure 1). *Implementation Risk*, as we understand it, is determined by three dimensions: (1) technical assessment of an AI system's robustness, (2) implementation feasibility for its intended clinical purpose and (3) risk evaluation of an AI system's potential harms and benefits.

**Figure 2:**
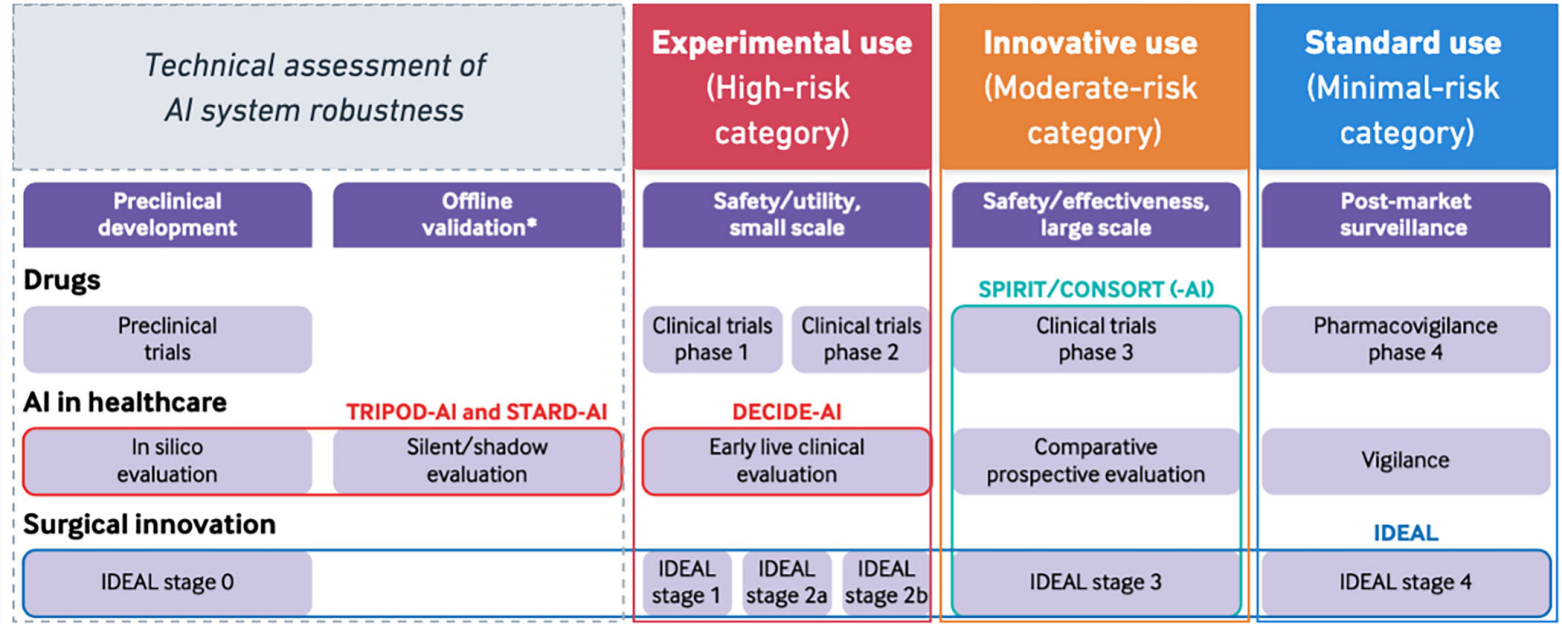
Recommendations for the clinical evaluation of AI systems based on *Implementation Risk* categorisation (adapted from Vasey et al. [13]) [32]. Stage 2 (Technical assessment of AI system robustness) may require in silico evaluation and silent/shadow evaluation (via TRIPOD‐AI and STARD‐AI guidelines), corresponding to preclinical trials for drug therapies and IDEAL stage 0 for surgical innovation. Experimental uses of AI require early live clinical evaluation to assess safety and utility (via DECIDE‐AI guideline), corresponding to phases 1 & 2 for clinical trials and IDEAL stage 1 to 2b. Innovative uses of AI require comparative prospective evaluation to assess safety and effectiveness (via SPIRIT or CONSORT‐AI guidelines), corresponding to clinical trials phase 3 and IDEAL stage 3. Standard uses of AI require post‐market surveillance, corresponding to the pharmacovigilance phase 4 and IDEAL stage 4.

Following its risk evaluation, an AI system is categorised as minimal‐risk (i.e., *standard use*), moderate‐risk (i.e., *innovative use*) or high‐risk (i.e., *experimental use*). Building on the DECIDE‐AI recommendations for early‐stage clinical implementation of AI [13], we propose proportional recommendations for implementation based on a model's *Implementation Risk* category (Figure 2). In general, higher risk categories (i.e., *innovative* and *experimental use*) require greater caution to deploy (i.e., more stringent human oversight, research ethics approval and rigorous clinical evidence), whilst minimal‐risk AI (i.e., *standard use*) may be implemented with only surveillance monitoring of its outputs and minimal human oversight.

The final consideration of our decision algorithm is to determine whether an AI system is within cost‐effective thresholds, and whether patients' informed consent is required before implementation of the system into patient‐facing clinical settings.

## 2 Implementation Risk Algorithm and Consent‐GPT Case Discussion

In the following section, we outline our decision algorithm based on an AI system's *Implementation Risk* and apply it to the case of Consent‐GPT, an LLM designed to seek surgical informed consent.

First, we look at the technical characteristics of an AI system to determine the scientific justification for its clinical application (**1. Technical assessment of system robustness**). Next, we explore any potential failure modes, which may be encountered during the clinical implementation and find practical strategies to address these (**2. Implementation feasibility**). Finally, we evaluate an AI system's risks and expected benefits, categorising it as either minimal‐risk (i.e., *standard use*), moderate‐risk (i.e., *innovative use*) or high‐risk (i.e., *experimental use*) (**3. Risk evaluation**).

Based on an AI system's risk category, we suggest that implementers (i.e., clinicians and hospital administrators) adopt different strategies for its clinical implementation (**4. Recommendations for clinical implementation**). Finally, in all cases, implementers should consider an AI system's cost‐effectiveness and the need for patients' informed consent (**5. Cost‐effectiveness threshold and Patient involvement**).

## 3 Technical Assessment of AI System Robustness [*Non‐Maleficence*]

Firstly, an AI system should be assessed to determine whether there is sufficient scientific justification for the technology to address its intended clinical purpose(s) (Box 3). Implementers are not required to provide extensive validation of a system's practical safety and effectiveness (this may not yet be known), but simply to establish a reasonable claim in favour of its clinical use. AI systems assessed as having no or very weak scientific justification for their intended clinical use(s) should be rejected at this stage, until new evidence arises.

Box 3 **Consent‐GPT—1. Technical assessment of system robustness.**

*Several studies may offer scientific justification for the proposed use of Consent‐GPT. For instance, a randomised controlled trial (RCT) has demonstrated that LLMs can deliver clinical information with a quality comparable to human physicians, notably enhancing patient understanding of the information required for informed consent* [14]*. Furthermore, LLMs have been positively evaluated for their readability* [15], *accuracy and comprehensiveness* [10, 16, 17], *and empathy* [18], *with certain studies highlighting their superior performance in generating responses to patient enquiries* [18, 19] *and documentation over human physicians* [16, 17]*. Additionally, the risk of “hallucinations” (i.e., fluent but false responses) may be minimised by training LLMs on larger, more semantically refined medical datasets* [20] *and by enhancing the neural models to interpret meaning at both the word‐level and context‐level of text inputs* [20]. *Accuracy of LLM‐based responses can be enhanced by retrieval augmented generators with domain‐specific knowledge databases and validated through human‐in‐the‐loop evaluations* [21].

*Thus, there seems to be a strong theoretical justification for the use of Consent‐GPT to seek patients' consent for surgery*.

This evidence may be provided by developers, deduced from specialised expert opinion (e.g., computer scientists, AI experts, specialised clinicians) or inferred from the existing scientific literature (e.g., animal models, physiological or biochemical rationale, observational research, audit, analytic reasoning, epidemiological evidence).

Evidence from clinical sources, while generally preferable, is not mandatory. Owing to the general‐purpose nature of many AI tools, there may be strong evidence supporting a system's robustness (i.e., functionality, limitations, risks) from a field outside of medicine (e.g., education, finance, engineering). In such cases, it may be more relevant for implementers to justify the specific clinical use(s) of the technology and its intended purpose, rather than simply the model's technical feasibility (which may already be apparent from evidence in other disciplines). This may also involve exploring additional considerations unique to the technology's clinical application.

## 4 Implementation Feasibility [*Non‐Maleficence/Justice*]

Next, implementers should consider potential failure modes, which may be encountered during a system's implementation into clinical practice (Box 4). The presence of these failure modes does not necessarily indicate that an AI system should be rejected. Rather, attempts should be made to mitigate their effects and to find alternative solutions if these failure modes are likely to cause serious barriers to implementation.

Box 4 **Consent‐GPT—2. Implementation feasibility.**

*Despite pre‐clinical evidence demonstrating the technical capabilities of an AI model like Consent‐GPT, there are still significant unknowns regarding the practical feasibility of its integration into clinical consent processes* [17, 21], *as well as the impact of (mis)trust in AI systems on public acceptance of their use* [22, 23]*. Such practical concerns may be described as potential failure modes of an AI application, and should be accounted for and addressed by clinicians prior to implementation, where possible*.

*One important consideration is the impact of biased or missing information in Consent‐GPT's training data, leading to some patient groups incurring disproportionate harms. For example, some studies suggest that LLMs may be designed with a bias towards high‐income countries, and perpetuate biases pertaining to race, sex, language and culture* [24, 25, 26].

*However, it may be possible to tractably resolve such biases through diversifying the training datasets of LLMs* [27]*. Moreover, it is worth noting that the information provided by human clinicians during the consent process is not always completely accurate or unbiased either* [28]*. It is therefore important that we set reasonable expectations for systems like Consent‐GPT when assessing its clinical feasibility. In fact, well‐designed AI algorithms have been shown to be promising tools for reducing racial disparities by minimising the risk of human subjectivity when interpreting data* [29]*. In this way, Consent‐GPT may perform better than human clinicians at providing unbiased information for patient decision‐making in consent*.

*Additionally, given the low burden of healthcare resources required for Consent‐GPT (e.g., patients could access Consent‐GPT via a digital application on their own personal device and in their own time), such a proposal would likely be easy to integrate without the need for extensive changes to existing clinical workflow or infrastructure*.

*Finally, clinicians will need to make sure that they comply with the relevant regulatory requirements before adopting this tool into clinical practice. While there are several examples of similar applications to Consent‐GPT in research settings* [14, 30, 31], *at the time of writing, no such AI tool has been granted market approval* (Box 6).

*However, as an LLM, Consent‐GPT would likely be classified as high risk by (some) regulators, requiring premarket approval and subject to additional transparency requirements to disclose the extent of its clinical use* [32].

Potential failure modes may be practical, ethical or legal (Box 5).

Box 5 **Potential failure modes for AI systems in medicine.**

***Practical*** [13, pp. 924–933]

- *Samples outside the distribution of training data*,
- *Different or outdated equipment that is not easily compatible with advanced AI technologies*,
- *Training and education for clinicians*,
- *Cost and resource constraints*,
- *Human factors (ergonomics) of integration into workflow*.

***Ethical*** [7, pp. 150–158]

- *A lack of interpretability and trust in AI‐generated recommendations*,
- *Biased data may result in some groups attracting disproportionate harms*,
- *The need for vast amounts of high‐quality data may be difficult to obtain due to privacy laws and data silos*,
- *Obtaining valid consent for AI to use patient data may be difficult, given the challenges in predicting future uses of the technology*,
- *The potential for misuse leading to large‐scale harm*.

***Legal*** [33]

- *A lack of clear and consistent regulatory guidance raises questions about legal responsibility for AI‐generated decisions*,
- *Rigorous clinical validation process (often via randomised controlled trials) required to prove efficacy and safety*.

*This list is non‐exhaustive, and there may be other considerations for potential failure modes specific to the AI technology or its intended clinical context*.

This stage also involves consideration of the relevant regulatory requirements for AI market approval. Specific requirements vary based on jurisdiction (Box 6), though they generally involve developers creating strategies to minimise the risks of bias, privacy leakage and AI system failures. Low‐risk applications can generally self‐certify, whilst higher‐risk applications will need to go through a process of regulatory approval.

Box 6 **Regulatory landscape for market approval of AI products in medicine in the USA, UK, EU, Australia and Singapore.**

***USA*** [34]

- *The FDA regulates AI/ML‐enabled software as a medical device (SaMD) based on its intended use and risk classification (Class I, II and III). High‐risk AI applications require rigorous pre‐market testing and approval to demonstrate safety and effectiveness*.
- *As of October 2023, the FDA has approved 692 AI/ML‐enabled medical devices, with over 80% approved since 2019 via the 510(k), De Novo, or premarket approval pathways. However, no devices relying on purely generative AI architectures have been approved yet*.
- *Consent‐GPT requirements: Would likely be regulated as a Class II or III device requiring premarket review to demonstrate safety and effectiveness. Human oversight, accuracy of outputs and transparency to patients would be key considerations*.
***UK*** [35]
- *The MHRA regulates AI as a medical device, requiring compliance with UK medical device regulations*.
- *MHRA has published guiding principles on good machine learning practices, transparency and predetermined change control for adaptive AI/ML*.
- *MHRA is collaborating with NICE on the regulation and evaluation of AI, especially for digital mental health*.
- *Consent‐GPT requirements: Would be regulated as a medical device with requirements for safety, efficacy, human oversight and transparency. MHRA may require a predetermined change control plan*.
***EU*** [32]
- *EU AI Act entered into force on Aug 1, 2024, and will be effective from Aug 2, 2026*.
- *AI Act categorises medical AI systems according to risk (minimal, moderate, high and unacceptable)*.
- *Additional considerations apply for generative AI systems (e.g., transparency requirements) and AI classified as a medical device (i.e., compliance with existing Medical Device Regulations (MDR) and In Vitro Diagnostic Medical Devices Regulation (IVDR)*.
- *Products with market approval receive a CE mark*.
- *Consent‐GPT requirements: Likely classified as high‐risk under the AI Act, requiring conformity assessment, human oversight, transparency and compliance with MDR/IVDR as a medical device. Generative AI transparency requirements would also apply*.
***Australia*** [36]
- *The TGA regulates AI‐enabled medical devices, including SaMD, based on risk classification. Devices require inclusion on the Australian Register of Therapeutic Goods*.
- *TGA has published guidance on the regulation of AI/ML medical devices and software more broadly*.
- *Large language models with a medical purpose supplied in Australia are regulated as medical devices*.
- *Consent‐GPT requirements: Regulated as a medical device demonstrating safety and performance. Must show quality of training data and relevance to the Australian population. Human oversight required*.
***Singapore*** [37]
- *The Health Sciences Authority regulates telehealth and software as a medical device*.
- *In 2021, Singapore published AI in Healthcare Guidelines (AIHGle), providing guidance on the development and implementation of medical AI*.
- *Consent‐GPT requirements: Likely regulated as a high‐risk telehealth/software medical device. AIHGle guidelines on clinical governance, transparency, human oversight and post‐deployment monitoring would apply*.

Once a system has been checked for its regulatory compliance and potential failure modes have been addressed or accounted for, implementers may move on to the next stage of clinical evaluation.

## 5 Risk Evaluation [Beneficence/Non‐Maleficence]

Finally (and crucially), implementers should determine whether the risks and expected benefits of implementing an AI system into clinical practice are justifiable (Box 7). This process involves establishing the value of potential harms and benefits associated with a system's clinical implementation, as well as ensuring fair distribution of potential harms and benefits within the intended clinical setting.

Box 7 **Consent‐GPT—3. Risk evaluation.**

*Consent‐GPT could offer considerable benefits to both patients and clinicians in the medical consent process. Not only could it improve patients’ access to the relevant information for decision‐making, but Consent‐GPT could also allow clinicians to focus their time on more complex tasks or spend longer with patients who specifically require human consent conversations*.

*However, there are also potential harms associated with Consent‐GPT. For example, patients may believe their views and feelings are undermined if they are made to interact with Consent‐GPT without the option of a human clinician, compromising the doctor‐patient relationship* [38].

*Additionally, patients may be harmed (i.e., physically, psychologically, emotionally) if Consent‐GPT were to provide them with inaccurate, incomplete or false information about a procedure's risks or complications. There may also be legal consequences for clinicians* [1, pp. 31–38].

*Consent‐GPT may also struggle to register patients’ non‐verbal cues, leading to incorrect assessments of patients’ valid consent (e.g., a patient lacks capacity, is perhaps under duress or does not understand the information)*.

*There may also be concerns about the potential persuasive effects of LLMs, like Consent‐GPT, to manipulate patient decision‐making (i.e., by tailoring its responses based on patients’ emotions or previous consent interactions)* [39]*. By influencing individuals’ decision‐making either inadvertently or by design, Consent‐GPT may compromise patients’ voluntariness, which is essential to truly informed consent. For example, in the case of clinical drug trials, pharmaceutical companies may have strong incentives to use LLMs for nefarious purposes, such as to subtly coerce or unduly influence patients’ decisions to participate* [1, pp. 31–38].

*It is also important to ensure the fair distribution of harms and benefits among patient groups if Consent‐GPT were implemented. For example, vulnerable populations with limited access to healthcare or lower digital literacy may be disproportionately affected, as they might struggle to effectively interact with or trust LLM‐based systems. These groups could miss out on the potential benefits of improved understanding and informed consent, leading to a widening of existing health disparities*.

*The risks associated with Consent‐GPT are not trivial. Nor are they insurmountable or ostensibly unacceptable to prohibit its implementation. Thus, analysis of the risks associated with Consent‐GPT (based on evaluation of the probability, nature and relative uncertainty of its harms and benefits) classifies it in the **moderate‐risk category (innovative use)***.

*In the case of Consent‐GPT, it seems reasonable that the expected benefits would likely outweigh the associated risks, and therefore, it should be considered for implementation*.

To evaluate the risks and expected benefits of an AI system, there are three key dimensions:

1. *Probability of Harms and Benefits*
2. *Nature of Harms and Benefits*
3. *Uncertainty of (1) and (2)*

ForHarms,(1)×(2)×(3)=Risk(R).

ForBenefits,(1)×(2)×(3)=ExpectedBenefit(EB).

The *Probability of Harms and Benefits* refers to the likelihood that a harm or benefit will occur as a result of using the AI system. Probabilities can be derived from clinical trials, historical data or expert opinion.

The *Nature of Harms and Benefits* refers to the severity or magnitude of an AI system's outputs. In theory, values of this metric could be determined either subjectively (i.e., by the patient's preferences) or objectively (i.e., in some other nonsubjective way, for example, via a specialised panel of experts). While there is disagreement among bioethicists regarding which approach may be most appropriate to fulfil ethical obligations of respect for autonomy and beneficence [40, 41, 42], it is not the intention of this paper to settle this debate.

Instead, we suggest a hybrid approach to evaluating the nature of harms and benefits. First, ‘objective’ values may be approximated based on how a ‘reasonable person’ would likely assess these consequences (i.e., ‘death’ as the worst possible outcome with the lowest value, etc.). Adjustments to these objective values can then be made through patient and public involvement and engagement (PPIE) focus groups, whereby key stakeholders provide direct feedback on these assigned values [43]. This approach ensures the perspectives and priorities of those affected by AI implementation are accurately incorporated.

The third dimension, *Uncertainty*, accounts for the level of confidence in the probability and nature of outcome estimates. Higher uncertainty indicates less confidence in the estimates, and can be due to limited data, low‐quality evidence or poor applicability of existing evidence to the AI's intended clinical use. Lower uncertainty (i.e., higher confidence) may be associated with larger sample sizes, more robust study designs (e.g., RCTs, prospective studies) and more clinically applicable evidence.

Thus, an AI system's net *Risk* and *Expected Benefit* is evaluated as the product of these three dimensions. We can then use this risk evaluation to categorise AI proposals as minimal‐risk (i.e., *standard use*), moderate‐risk (i.e., *innovative use*) and high‐risk (i.e., *experimental use*). We have intentionally refrained from assigning definitive numerical values to each of these risk categories to maintain flexibility and account for the nuanced and context‐specific nature of AI applications in medicine.

Describing an intervention or clinical process as *innovative* or *experimental use* identifies it as deviating in some normative way from existing conventional practice.^1^ In contrast, *standard use* signifies that an intervention does not introduce any additional risk beyond what is already accepted in current practice. The difference between *innovative* and *experimental use* reflects the degree of departure from traditional clinical processes (i.e., innovative: *moderate* departure; experimental: *significant* departure) and thus, the level of uncertainty regarding the AI's safety, efficacy and ability to integrate into practice [45].

Once the risk category has been determined, implementers should weigh up the risks against the expected benefits of an AI proposal. This deliberation process will likely require implementers to make normative trade‐offs about which expected benefits or risks should carry greater moral significance. Rather than propose a specific approach to weighing up potential outcomes of an AI system, we suggest that implementers adopt a process of deliberative review, whereby potential outcomes are evaluated during round‐table discussions among the implementing team of clinicians.

During this process, it is also important that potential harms and benefits are fairly distributed among potential patient groups to ensure that certain groups do not attract disproportionate harms from the use of AI. In some circumstances, it may be appropriate to consider selective deployment (i.e., only where the AI would clearly benefit) [46]. This approach ensures that potential inequities can be addressed and mitigated before wider implementation.

There are three possible outcomes when weighing up the expected benefits and risks of an AI proposal. Firstly, if the risks outweigh the expected benefits, the proposal should be rejected until there is further evidence to support its use. Alternatively, proposals for which the expected benefits clearly outweigh the risks should be considered for implementation. Finally, there may be some AI proposals for which it is unclear whether the expected benefits outweigh the risks. In such circumstances, we recommend clinicians seek a wide range of expert opinion (i.e., IT experts, AI ethicists), including those from different countries and with differing values. Where there is consensus against an AI system's use, the proposal should be rejected. Where there is dissensus (i.e., reasonable disagreement) [44] among experts in favour of the expected benefits outweighing the risks, the proposal should be considered for implementation so long as expert opinion remains equivocal. This approach ensures safe innovation and scientific exploration to improve understanding of an AI tool's risks and benefits [1, pp. 31–38]. A similar process is also used in the case of innovation in surgical techniques, whereby surgeons are permitted to implement novel techniques within certain ethical and regulatory frameworks [47].

## 6 Recommendations for Clinical Implementation

Depending on the risk category, we suggest different recommendations for implementation (Box 8).

Box 8 **Consent‐GPT—4. Recommendations for implementation.**

*Consent‐GPT is categorised as an innovative use (moderate‐risk category). As such, clinicians need to seek approval from the hospital's research ethics committee (REC) before its clinical implementation*.

*They also need to establish a process of regular human oversight of Consent‐GPT's performance. This may involve an initial period of rigorous oversight, whereby every consent interaction using Consent‐GPT is evaluated and the patient's consent is then reviewed by the treating surgeon. However, after this initial intensive stage, it may be appropriate to reduce the human oversight and review process to only those consent interactions, which have been flagged as challenging for the AI (i.e., by Consent‐GPT, by reviewing clinicians or self‐identified by the patient)*.

*Finally, as an innovative use of AI, Consent‐GPT's performance should be evaluated for its clinical safety and effectiveness via comparative prospective evaluation, such as an RCT or prospective cohort study. The aim of this comparative assessment is to determine whether the medical consent process using Consent‐GPT is equivalent (or superior) to conventional methods for consent*.

AI classified as *experimental* and *innovative uses* should be required to apply for approval from the relevant research ethics committee (REC). As part of their REC application, clinicians should provide information relating to the technical assessment (i.e., proof of concept), implementation feasibility (including strategies to mitigate potential failure modes) and risk evaluation (including information regarding the potential risks and the expected benefits, as well as any relevant expert opinion). In contrast, *standard use* AI we believe could be exempt from REC approval and may be implemented directly.

Similarly, *experimental* and *innovative uses* of AI require human‐in‐the‐loop processes to ensure human oversight of the AI's outputs. This process not only ensures that the AI is technically performing as intended, but also that the expected benefits continue to outweigh the potential risks and to prevent or minimise the effects of potential harms.

Finally, we recommend that additional clinical evidence be collected relative to the AI's risk category. Accordingly, we build on existing recommendations by the DECIDE‐AI Steering Group for the early clinical evaluation of AI‐based decision support systems (Figure 2). However, where the DECIDE‐AI group propose a linear process of AI evaluation based on a system's developmental stage, we incorporate a third dimension for consideration based on the AI's risk category. This risk‐based approach is complementary to existing regulatory frameworks for AI governance [32], and supports the smooth transition from regulatory approval to successful implementation into clinical practice.

Thus, *experimental use* of AI should be evaluated for its technical safety, clinical utility and human factors. These AI systems tend towards a higher magnitude of harm and greater uncertainty in their potential risks and expected benefits. Therefore, they require a greater degree of caution during their clinical evaluation. Examples of appropriate study designs for *experimental use* AI include prospective cohort studies and non‐RCTs. Relevant reporting guidelines include the TRIPOD‐AI and STARD‐AI guidelines for offline validation of the AI system, and the DECIDE‐AI guideline for early live clinical evaluation.

*Innovative use* of AI should be evaluated for its clinical safety and effectiveness. These AI proposals do not need to go through the preceding evaluation phases for *experimental use* AI (i.e., preclinical development, offline validation, safety/utility assessment). Instead, these systems can proceed directly to comparative prospective evaluation (e.g., via RCT). The purpose of this evaluation is to improve the certainty of the AI system's potential harms and benefits for risk evaluation, as well as compare its performance against existing methods.

Finally, *standard use* AI may proceed directly to post‐market surveillance. The aim of this evaluation is to maximise benefits whilst monitoring for potential harms. These AI systems should be designed with automatic or ongoing self‐auditing processes to evaluate outcomes and ensure the system continues to meet existing standards for clinical care.

All risk categories require a process of regular monitoring and iterative review. This is to ensure that the AI's risk categorisation remains up‐to‐date as technology and clinical standards evolve over time.

## 7 Cost‐Effectiveness Threshold and Patient Involvement [*Distributive Justice and Respect for Autonomy*]

There are two additional ethical considerations relevant to all AI risk categories (Box 9).

Box 9 **Consent‐GPT—5. Cost‐effectiveness threshold and patient involvement.**

*Cost‐effective analysis will vary depending on healthcare systems, institutional funding and the degree of benefit provided by the intervention. While it seems plausible that Consent‐GPT would likely be within cost‐effectiveness thresholds (given its inherently low cost and minimal burden on healthcare resources), we cannot accurately predict this until we know how effectively it improves the surgical consent process. Somewhat paradoxically, this requires clinical implementation of Consent‐GPT. In the interim, it may be appropriate to use the analysis of Consent‐GPT's risks and expected benefits calculated in **Step 3** as a proxy for effectiveness*.

*Additionally, as a tool for seeking patients' consent for surgery, Consent‐GPT would be required to interact directly with patients. Therefore, Consent‐GPT should be offered as an option (either in addition to, or instead of, typical consent conversations), and patients can choose to validly consent or refuse and interact with a human clinician instead*.

The first is to determine whether the implementation of the AI system into the existing clinical workflow is within the cost‐effective threshold [48]. If not, then it may be necessary to consider alternative sources of funding. Decisions to implement will therefore be dependent on whether funding is available and the presence of competing candidates.

The second consideration is whether the patient will engage directly with the AI system, or whether the system is non‐patient‐facing (i.e., intended for administrative purposes). In the case of the former, the AI proposal should be offered to patients so that their values and preferences can be considered. This is also an opportunity to express to the patient the clinical confidence in the AI system based on its scientific justification and risk evaluation. The patient may then choose to validly consent, or refuse and seek standard or no care. If the system is non‐patient‐facing, then it may be implemented directly, in accordance with recommendations from *Step 4*.

## 8 Discussion

Our decision algorithm builds on previous work by the DECIDE‐AI Steering group [13, pp. 924–933] by proposing that clinical implementation of an AI system should be determined by an assessment of its risk. Thus, our algorithm provides proportionate recommendations for clinical evaluation, human oversight and ethics review. Higher risk categories (i.e., *experimental* and *innovative uses of* AI) should be implemented with a greater degree of caution (including human oversight and REC approval), whilst low‐risk (i.e., *standard use*) AI should aim to maximise clinical benefits through direct implementation with a follow‐up review to identify and prevent potential harms.

Our algorithm is the first to offer an ethical risk‐based approach to the safe and effective implementation of novel AI systems in medicine. It reflects recent changes to AI governance policy in the EU, UK and US, which stratify AI regulations based on a system's risk classification (specific requirements vary by jurisdiction) [32, 35, 49].

While we have explored in detail the case of Consent‐GPT (which we classify as moderate‐risk, i.e., *innovative use)*, Box 10 applies our algorithm to a list of other clinical examples of AI of varying risk categories.

Box 10 **List of additional examples of AI in medicine according to *implementation risk* categorisation.**

**Table:** 

*Minimal‐risk (i.e., standard use)*	*Moderate‐risk (i.e., innovative use)*	*High‐risk (i.e., experimental use)*
**LLMs to write patient discharge summaries** [50] 1. ***Technical assessment:** evaluation of consistency, factual accuracy and adherence to medical guidelines*. 2. ***Implementation feasibility:** consider integration into existing hospital systems, user interfaces and ease of use by healthcare staff*. 3. ***Risk evaluation**:* ▪ *Benefits—Improves efficiency and reduces administrative burden on healthcare providers. Ensures consistency and accuracy in discharge summaries*. ▪ *Harms—Possible inaccuracies or omissions in the summaries. Misinterpretation of the AI‐generated text by patients or healthcare providers* 4. ***Recommendations:** implement with minimal human oversight, regular monitoring and audits to ensure accuracy and reliability*. **AI in diagnostic image analysis** [51] 1. ***Technical assessment**: assess the accuracy of image interpretation compared to human radiologists and standard benchmarks*. 2. ***Implementation feasibility**: ensure compatibility with existing imaging equipment and workflows in radiology departments*. 3. ***Risk evaluation**:* ▪ *Benefits—Speeds up diagnostic processes and reduces human error. Can handle large volumes of images quickly and consistently*. ▪ *Risks— Misdiagnoses due to AI errors or biases. Over‐reliance on AI by healthcare providers, potentially missing rare conditions*. 4. ***Recommendations**: implement with regular monitoring and audit processes by IT specialists and radiologists*.	**LLMs to assess clinical acuity in emergency departments** [52] 1. ***Technical assessment:** evaluate the accuracy and reliability of the AI in prioritising patients based on severity*. 2. ***Implementation feasibility:** ensure seamless integration into emergency department workflows and training for healthcare providers*. 3. ***Risk evaluation:*** ▪ *Benefits— Optimises resource allocation in emergency departments. Potentially improves patient outcomes by prioritising those in greatest need*. ▪ *Risks—Incorrect triage decisions could delay critical care for some patients. Over‐reliance on AI might reduce the quality of human clinical judgement* 4. ***Recommendations:** requires REC approval, regular human oversight and comprehensive evaluation of clinical safety and effectiveness through comparative studies*. **AI in liver allocation for transplant** [53] 1. ***Technical assessment:** evaluate the algorithm's decision‐making process, including fairness, bias and transparency in the allocation decisions*. 2. ***Implementation feasibility:** ensure alignment with existing organ allocation policies*. 3. ***Risk evaluation:*** ▪ *Benefits— Potentially improves fairness and efficiency in organ allocation. Can process complex data to make informed allocation decisions*. ▪ *Risks—Life‐or‐death consequences if the AI makes incorrect allocation decisions. Ethical concerns about fairness and transparency in the decision‐making process* 4. ***Recommendations:** Requires continuous human oversight, rigorous evaluation through prospective studies and approval from research ethics committees (REC)*.	**AI for embryo selection** [9] 1. ***Technical assessment:** validate the AI's accuracy in predicting viable embryos, accounting for potential biases and error rates*. 2. ***Implementation feasibility:** consider the impact of dynamic biological processes on AI prediction accuracy and the ability to integrate into existing IVF workflow and infrastructure*. 3. ***Risk evaluation:*** ▪ *Benefits— Improves the selection process for viable embryos, potentially increasing success rates in IVF. Reduces human bias and error in the selection process*. ▪ *Risks—Incorrect selection could negatively affect pregnancy outcomes. Ethical and social implications regarding the selection criteria used by AI*. 4. ***Recommendations:** requires continuous oversight, REC approval and further clinical evaluation through non‐randomised control trials*.

A key strength of this decision algorithm is its ability to adapt to changes in AI technology and clinical practice. Focusing on an AI system's risks means that recommendations for its implementation can adapt to new information about a model's safety and effectiveness. For example, initially, a system might be classified as *standard use* AI (i.e., minimal risk). As the system updates and modifies over time, new clinical evidence may suggest re‐evaluation of its risk category (i.e., changes to the probability, nature and/or uncertainty of its potential harms and benefits). Thus, a *standard use* AI may shift to *innovative* or *experimental use*, and so require additional considerations for implementation. This re‐evaluation process requires ongoing monitoring and review of an AI system's outputs to ensure risk categorisations are up‐to‐date.

Our decision algorithm also emphasises proportional recommendations for human‐in‐the‐loop processes, such as clinical evaluation, human oversight and ethics review. This approach allows scarce healthcare resources (particularly those requiring human clinical input) to be allocated more efficiently based on the AI system's risk level. Additionally, our algorithm also considers the cost‐effectiveness of implementing an AI system clinically, ensuring that resources are distributed equitably within a healthcare system.

However, there are also important limitations to our decisional algorithm, which should be considered.

Firstly, it may be difficult to accurately evaluate an AI system's risks and expected benefits. There may be a number of reasons for this. For example, there may be a lack of historic data about existing (human) processes to compare and validate an AI's clinical performance. Or, it may be difficult to determine which metrics should be used for clinical evaluation (particularly in the case of general‐purpose AI, where a system can perform multiple different tasks, which it may not have been specifically designed for) [54, 55].

This concern presents an ongoing challenge for regulators and clinicians alike, especially given that market approval in many jurisdictions globally is dependent on risk classification [56]. However, it seems likely that clinical evaluation of these systems will improve over time as they become more commonplace in clinical practice. Until then, a proxy human clinician standard may be used as an appropriate benchmark to evaluate a model's risk and expected benefits.

There may also be more general concerns about the inherent lack of interpretability of black‐box AI systems, preventing some forms of clinical evaluation (e.g., assessing the risks associated with *how* a black‐box AI system arrives at certain clinical decisions). Some have argued in favour of interpretable AI systems as an essential requirement for their safe and effective implementation into clinical practice [9].

In contrast, our algorithm supports the clinical implementation of AI systems without specific requirements for transparency or interpretability of its processes. This is because an explanation of how an AI system operates does not necessarily provide justification for its use. Indeed, there are many medical interventions (e.g., statins, paracetamol) that are used without understanding how they work. While explainability may influence the extent to which patients trust AI systems, we argue that clinical implementation should be justified based on the risks associated with its outputs [7, pp. 150–158, 57, 58]. This is determined by robust clinical evidence supporting a model's reliable performance.

## 9 Conclusion

We have argued that decisions about whether or when to implement AI‐based tools in medicine should be based on a model's implementation risk (rather than design‐specific factors, such as interpretability). Thus, we propose a practical decision algorithm for the ethical implementation of black‐box AI systems in medicine. Our algorithm aligns with recent changes in the regulations of AI systems, which now adopt a risk‐based approach.

By suggesting proportional recommendations for human oversight, ethics review and clinical evaluation, we aim to support clinicians to implement AI systems into clinical practice ethically and responsibly. Such decision‐making support is essential for the widespread adoption of these tools in medicine, as it fosters technical innovation whilst still maintaining patient safety.

## Data Availability

Data sharing is not applicable to this article as no data sets were generated or analysed during the current study.

## Endnotes

1 We recognise that the terms ‘innovative’ and ‘experimental’ are used extensively throughout the bioethical and medical literature with varying definitions, and sometimes interchangeably to mean the same thing. See Wilkinson & Savulescu [44]. Papastefan et al. [45], pp. 1609–1612). In this paper, we strictly refer to these terms as they are described here (i.e., to provide a normative classification for the implementation of different AI tools).
